# Changes in Oral Health Knowledge, Beliefs, and Self-Reported Practices Among Preschool Teachers Between 3 and 12 Months After School-Based Education

**DOI:** 10.3390/dj14100662

**Published:** 2026-10-12

**Authors:** Gunes Koc, Ozgul Baygin

**Affiliations:** Department of Pediatric Dentistry, Faculty of Dentistry, Karadeniz Technical University, Trabzon 61080, Turkey

**Keywords:** preschool teachers, oral health education, knowledge retention, longitudinal study, fluoride, dental trauma, health promotion

## Abstract

**Background/Objectives:** Preschool teachers can reinforce oral health practices, but the persistence of educational messages requires evaluation. This study assessed changes in oral health knowledge, beliefs, and self-reported practices between 3 and 12 months after school-based education. **Methods:** Education reached 240 teachers in 63 schools in Trabzon, Turkey. Questionnaires were completed by 222 teachers at 3 months and 152 at 12 months. Paired analyses included the 152 teachers assessed at both follow-ups. Responses were compared using McNemar tests. No matched pre-education assessment or concurrent control group was available. **Results:** The paired cohort represented 63.3% of trained teachers. Endorsement of fluoride-containing products for caries prevention decreased from 63.8% to 40.1%, recognition of enamel strengthening from 82.9% to 66.4%, and identification of first-tooth eruption as the time to begin cleaning from 92.8% to 75.7% (all *p* < 0.001). Recognition that avulsed primary teeth should not be replanted decreased from 83.6% to 70.4% (*p* < 0.001), whereas endorsement of immediate permanent-tooth replantation changed little (91.4% versus 89.5%; *p* = 0.508). Twice-daily brushing decreased from 82.9% to 73.0% (*p* = 0.001). Tests were exploratory and unadjusted for multiple comparisons. **Conclusions:** Between 3 and 12 months, several preventive messages were less frequently endorsed, and some self-reported practices changed. These findings identify topics for updated content and refresher education concerning fluoride, early oral care, primary-tooth avulsion, and routine preventive dental visits. The observations do not establish initial educational effectiveness or clinical benefit in children.

## 1. Introduction

Preventing oral disease in early childhood requires consistent support across the settings in which children live and learn. The World Health Organization’s global oral health report identifies a continuing need to integrate prevention into wider health and social systems [1]. Dietary sugar exposure and daily use of fluoride toothpaste are central to caries prevention, supported by systematic reviews of sugar restriction and fluoride toothpaste effectiveness [2,3]. Age-appropriate advice is particularly important during the transition from the eruption of the first teeth to increasingly independent self-care [4]. Preschool teachers are well placed to reinforce these messages through everyday routines and communication with families.

The health-promoting school approach extends beyond transmitting information. It connects staff capabilities with school policies, the learning environment and family participation [5]. A systematic review covering preschool through secondary education found generally favourable outcomes from school oral health programmes, particularly those involving children, teachers and parents [6]. Nevertheless, educational improvement should not be equated with clinical benefit. Stein et al. found that conventional school oral health education could reduce plaque, but evidence for preventing caries and sustaining long-term clinical effects was insufficient [7]. Evaluation therefore needs to distinguish teachers’ learning, the maintenance of that learning, its practical application and children’s subsequent oral health.

Teachers’ baseline knowledge is also uneven across topics and settings. In Turkey, Yilmaz et al. reported a weak relationship between perceived and measured knowledge and identified deficiencies in dental trauma management [8]. A survey in Nepal similarly identified a need to strengthen teachers’ oral health knowledge [9]. More recently, Wang et al. examined oral health knowledge, attitudes and practices among parents and kindergarten teachers, highlighting the relevance of both groups to the child’s preventive environment [10]. These studies support assessing training needs directly rather than assuming that professional experience or confidence ensures adequate preventive knowledge.

The present study examined preschool teachers within a school oral health education programme in Trabzon, Turkey. Its contribution is the paired assessment of knowledge, beliefs and self-reported practices at 3 and 12 months after education. Following the same teachers allows topic-specific changes to be described over time.

Teacher training is feasible in different delivery settings. Fernando et al. evaluated a preschool intervention in Sri Lanka, and Dedeke et al. reported improved knowledge following a teacher-training workshop in Nigeria [11,12]. A large Hong Kong programme documented high satisfaction and perceived improvements in kindergarten teachers’ knowledge and competence after training [13]. These outcomes are encouraging, but the immediate response to education does not establish whether particular messages remain accessible months later. A trial of school oral health education in adolescents also showed the importance of repetition and reinforcement, although its findings cannot be transferred directly to preschool teachers’ knowledge retention [14].

Accordingly, this study examined changes in item-level oral health knowledge, beliefs and self-reported personal practices among the same teachers between 3 and 12 months after education. It sought to identify messages that remained relatively stable and those that might require reinforcement. The analysis describes post-education change within the programme.

## 2. Materials and Methods

### 2.1. Study Design and Programme Continuity

This single-group longitudinal study evaluated preschool teachers at 3 and 12 months after school-based oral health education in the Ortahisar district of Trabzon, Turkey. The schools were revisited to reassess teachers who had received the education, using the same questionnaire at both follow-ups. The analysis was restricted to teachers with responses at both time points.

No individually matched pre-education measurements were available for the present analysis, and no concurrent comparison group was included. Reporting follows relevant domains of the Strengthening the Reporting of Observational Studies in Epidemiology statement and the Template for Intervention Description and Replication checklist [15,16].

### 2.2. Setting and Participants

All 63 participating schools were public primary schools with preschool classes in Ortahisar, Trabzon. Participants were the teachers of these preschool classes. School names and addresses were obtained from the education authorities. The district sampling frame comprised 306 preschool teachers, of whom 240 were reached in 63 schools and received education. At 3 months, 222 teachers completed the questionnaire. At 12 months, 152 teachers who were available and agreed to participate completed it again. Their corresponding 3-month responses were used for paired comparisons. The Provincial Directorate of National Education administrative record concerning permission for the study is dated 26 October 2017. The follow-up investigation commenced on 19 November 2017, following ethics approval. Assessments took place at schools during teachers’ working hours, at 3 and 12 months after education; exact calendar dates for individual educational sessions and assessments were not available in the supplied records.

Participation was voluntary. Teachers who were absent or declined to complete a follow-up questionnaire did not contribute data at that assessment. The paired analysis used complete follow-up cases, with no imputation for teachers lost to follow-up. Recruitment was based on access to the eligible school population; a prospective sample-size calculation was not reported. Characteristics of non-respondents were not available for comparison with those retained. The available records did not contain comparable individual characteristics or item-level responses for the 70 teachers who completed the 3-month assessment but not the 12-month assessment. A comparison of retained and lost participants was therefore not possible.

### 2.3. Educational Intervention

The educational component was delivered by the research dentist E. Baltaci, and follow-up assessment was undertaken within G. Koc’s subsequent thesis investigation. The programme, entitled “Oral and Dental Health in Children,” used face-to-face, computer-assisted presentations. Sessions were held in classrooms, seminar rooms, staff rooms or meeting rooms according to each school’s facilities. The follow-up analysis reported here was undertaken as part of G. Koc’s 2020 specialty thesis and used teachers previously reached by the educational programme described by Baltaci et al. [17]. The present manuscript reports the paired 3- and 12-month follow-up data from that thesis.

Content covered teachers’ role in supporting oral health; primary and permanent dentition and tooth eruption; the consequences of oral disease; plaque, calculus and caries; dietary practices; oral hygiene for infants and children; fluoride in caries prevention; non-nutritive sucking habits; and the management of traumatic dental injuries. A common presentation framework was used across the participating schools. Session duration, the number of contacts per teacher and formal checks of delivery fidelity were not specified in the available programme documentation. Exposure to additional education between follow-ups was not systematically recorded.

### 2.4. Questionnaire and Assessment Procedures

The questionnaire contained three sections addressing demographic characteristics, general oral health knowledge, and oral health attitudes and practices. It included open-ended, closed-ended and multiple-response questions and drew on the instrument used in the earlier study [17]. That instrument had been piloted with 10 teachers to assess comprehensibility. The follow-up questionnaire was not established as a psychometrically validated knowledge scale, and no composite knowledge score was calculated.

Teachers completed the questionnaires at school during working hours, taking approximately 10 min. The investigator remained at a distance and clarified questions when requested. Demographic information included sex, age, parental status and professional experience. Outcomes were the proportions selecting individual responses concerning caries, prevention, early oral care, fluoride, dental trauma, sucking habits and personal oral health practices. For questions allowing several answers, each response option was considered separately.

Knowledge items were distinguished from beliefs and self-reported practices. In particular, the historical statements describing fluoride as “nutritive for teeth and bones” and recommending initiation of fluoride toothpaste around 3 years of age were retained as questionnaire endorsements, not scored as correct contemporary advice. This distinction is necessary because current guidance supports age-appropriate fluoride toothpaste from first-tooth eruption [4]. Primary- and permanent-tooth avulsion were assessed separately in view of their different management recommendations [18,19].

### 2.5. Statistical Analysis

The original study analysis used SPSS version 17.0 for Windows (SPSS Inc., Chicago, IL, USA). Counts and percentages describe participant characteristics and item-level responses. Paired responses at 3 and 12 months were compared using McNemar tests, with *p* < 0.05 as the nominal significance threshold. The earlier analysis also included exploratory chi-square comparisons by parental status and professional experience.

Percentages were calculated using the 152 paired participants as the denominator, and absolute changes were expressed as 12-month minus 3-month percentages in percentage points. Inferential results are those recorded in the original thesis report; *p* values were not recalculated from marginal totals. Individual response-transition counts were not available for independent reanalysis or calculation of paired confidence intervals. Tests were not adjusted for multiple comparisons or clustering within schools and are interpreted as exploratory. For outcomes without a reported paired test, only descriptive differences are presented.

### 2.6. Ethics and Participation

Approval was obtained from the Scientific Research Ethics Committee of Karadeniz Technical University Faculty of Medicine (protocol 2017/198; decision 20, dated 13 November 2017; notification dated 19 November 2017). Permission for school visits and questionnaire administration was obtained from the education authorities; the related Provincial Directorate of National Education administrative record is dated 26 October 2017. Teachers were informed about the study, provided written informed consent and participated voluntarily.

### 2.7. Manuscript Preparation

ChatGPT (GPT-5, OpenAI), accessed through a ChatGPT Plus subscription, assisted with drafting, English-language revision, organisation, literature identification, and formatting using the supplied study documents. Bibliographic records were checked against source records. The reported observations and original inferential results were retained; no participant-level data were generated using artificial intelligence. Scientific verification and final approval remained the responsibility of the authors.

## 3. Results

### 3.1. Participant Flow and Characteristics

The programme reached 240 teachers in 63 schools. At 3 months, 222 teachers participated, corresponding to 92.5% of those trained. The paired cohort of 152 represented 63.3% of the initial group and 68.5% of the 3-month respondents. Thus, 70 teachers who completed the 3-month assessment were not retained in the paired analysis. The final sample comprised 133 women (87.5%) and 19 men (12.5%); 112 teachers (73.7%) had children and 77 (50.7%) had more than 10 years of professional experience (Table 1).

### 3.2. Caries and Preventive Messages

At 3 months, most teachers endorsed inadequate brushing and sweet or acidic foods as contributors to caries. Endorsement of sweet or acidic foods remained high at 12 months (98.7% versus 96.7%; *p* = 0.375). In contrast, endorsement of plaque decreased from 84.2% to 71.1%, and that of the absence of regular check-ups decreased from 84.9% to 63.2% (both *p* < 0.001). The proportion believing that caries-free teeth were possible decreased from 94.1% to 79.6%, while recognition that primary-tooth caries could be treated remained high (98.0% versus 96.7%; *p* = 0.625).

Among the caries-prevention options, endorsement of fluoride-containing dental products decreased by 23.7 percentage points, from 63.8% to 40.1%. Endorsement of regular dental visits decreased by 22.4 points, from 84.2% to 61.8%, and that of interdental flossing decreased by 14.5 points, from 92.8% to 78.3% (all *p* < 0.001). Endorsement of limiting sugary snacks remained relatively high (95.4% versus 92.8%; *p* = 0.388). Full item-level results, including non-significant comparisons, are presented in Table 2.

### 3.3. Early Oral Care and Fluoride

Recognition that tooth cleaning should start when the first tooth erupts decreased from 92.8% to 75.7% (*p* < 0.001). The proportion selecting a first dental examination no later than 1 year of age decreased from 85.5% to 76.3% (*p* = 0.013). Endorsement of fluoride strengthening enamel decreased from 82.9% to 66.4%, and endorsement of its ability to improve initial carious lesions decreased from 77.0% to 56.6% (both *p* < 0.001).

The proportion endorsing initiation of fluoride toothpaste at approximately 3 years decreased from 64.5% to 54.6% (*p* = 0.028). Because this response is not aligned with current preventive guidance, the decrease cannot be interpreted as loss of correct knowledge. Separately, the proportion stating that children should not use fluoride toothpaste increased from 20.4% to 29.6%; no paired *p* value was reported for that response. These historical beliefs and the early-care items are shown in Table 3.

### 3.4. Dental Injuries and Sucking Habits

Recognition that an avulsed primary tooth should not be replanted decreased from 83.6% to 70.4% (*p* < 0.001). Endorsement of prompt assessment of oral injuries also decreased, from 92.8% to 87.5% (*p* = 0.021). In contrast, endorsement of immediate replantation of an avulsed permanent tooth changed little (91.4% versus 89.5%; *p* = 0.508). Recognition that non-nutritive sucking habits can affect the teeth and jaws was 96.7% at both assessments (*p* = 1.000). These are questionnaire responses; practical emergency-management performance was not assessed (Table 3).

### 3.5. Personal Practices and Attitudes

Self-reported brushing at least twice daily decreased from 82.9% to 73.0% (*p* = 0.001), and reported toothbrush replacement at fixed intervals decreased from 90.8% to 80.3% (*p* = 0.004). Changes in brushing duration and interdental cleaning did not reach nominal statistical significance. Routine dental attendance without pain or another problem remained uncommon at both follow-ups: 16.4% at 3 months and 15.8% at 12 months (*p* = 1.000). Teachers’ belief that dentists sufficiently prioritised prevention increased from 60.5% to 75.7% (*p* = 0.001; Table 4).

Lack of time was reported as a reason for avoiding or disliking dental attendance by 19.7% at 3 months and 35.5% at 12 months. The proportions reporting cost or fear were lower at the later assessment. These distributions are descriptive because paired tests were not reported. One response was missing at 12 months (Table 5).

### 3.6. Exploratory Subgroup Findings

Most comparisons by professional experience and parental status were not statistically significant in the original analysis. Teachers with children were less likely than those without children to endorse fluoride-containing dental products at both assessments (58.0% versus 80.0% at 3 months, *p* = 0.013; 33.0% versus 60.0% at 12 months, *p* = 0.003). These cross-sectional subgroup contrasts do not establish different longitudinal rates of change. They were unadjusted and should be viewed as hypothesis-generating.

## 4. Discussion

### 4.1. Principal Findings and Contribution of the 12-Month Follow-Up

The central finding is that preschool teachers’ responses did not follow a uniform pattern during the year after education. Endorsement of several preventive messages declined between 3 and 12 months, particularly those concerning fluoride-containing products, early tooth cleaning, regular dental attendance and avoidance of replanting an avulsed primary tooth. Other responses, including recognition of the role of sugary snacks and the treatability of caries in primary teeth, remained comparatively stable. This topic-specific pattern is more informative for programme revision than a single overall knowledge score.

The paired observations show which messages remained endorsed and which became less frequently selected during follow-up. The longitudinal evidence concerns the 3- to 12-month interval. Without matched pre-education responses, the magnitude of initial learning cannot be estimated, and a decline between follow-ups does not establish that knowledge returned to its original level.

The results also show why initial uptake and sustained learning should be evaluated separately. At 3 months, recognition of several preventive messages was high, yet this did not consistently persist to 12 months. Ceiling effects may partly explain stability for some familiar items, while less intuitive or less frequently used information may require repeated application. These are plausible interpretations of the pattern, rather than mechanisms directly tested in this study.

### 4.2. Learning Retention and Reinforcement

Teacher-training evaluations in Sri Lanka and Nigeria support the feasibility of improving oral health knowledge through educational contact [11,12]. The Hong Kong programme further shows that teachers value training and perceive gains in their capacity to deliver oral health education [13]. Its post-training satisfaction and perceived-competence measures, however, assess a different outcome from repeated item-level responses. Our findings complement such evaluations by examining what happens after the initial educational period.

Evidence from sustained school programmes suggests that repetition can be part of a broader preventive system. Rong et al. combined recurring teacher, child and parent education with supervised fluoride toothbrushing over two years and reported a lower caries increment in the intervention group [20]. Because the programme contained several components, its clinical benefit cannot be attributed to teacher education alone. In Wuhan, a three-year health-promoting school programme improved several behavioural and gingival outcomes and teachers’ knowledge, but did not demonstrate a reduction in caries incidence [21]. These contrasting outcomes underline the importance of measuring both implementation and the clinical consequences of education.

Haleem et al.’s trial provides more direct evidence for examining repetition and reinforcement in school oral health education [14]. In the current programme, the decrease in endorsement of selected messages provides a rationale for testing refresher contacts, but does not identify an optimal timetable. A future comparison could evaluate scheduled reinforcement against the existing educational approach, with prespecified outcomes at several intervals. Brief retrieval questions, case discussions and opportunities to practise explaining preventive advice could be assessed as feasible additions to teachers’ routines.

### 4.3. Prevention Content and the Meaning of Response Changes

Responses concerning sugar restriction were relatively stable, whereas endorsement of fluoride-containing products declined substantially. Sugar reduction and fluoride toothpaste have distinct but complementary preventive roles [2,3]. A programme that communicates one message successfully may still leave uncertainty about the other. The contrast suggests a need to assess specific concepts and misconceptions, rather than assuming that a generally favourable attitude towards prevention implies understanding of all recommended practices.

The fluoride findings require particular care. Less frequent recognition of enamel strengthening and caries prevention is concerning, but reduced endorsement of an outdated or ambiguous statement cannot automatically be called loss of correct knowledge. The questionnaire’s wording about starting fluoride toothpaste around age 3 does not reflect current European Academy of Paediatric Dentistry guidance [4]. Similarly, describing fluoride as nutritive for teeth and bones does not capture its principal preventive action. Reporting these items transparently as historical beliefs preserves the observations while avoiding an inappropriate correctness score.

Updated educational materials should distinguish when toothbrushing begins, the amount and concentration of toothpaste, adult supervision and fluoride’s preventive purpose [3,4]. The fall in endorsement of first-tooth eruption as the starting point for cleaning suggests that practical timing deserves emphasis. Yet the questionnaire cannot establish whether changed answers reflected forgetting, exposure to other information or a different interpretation of the item. Future instruments should use clearly worded, guideline-consistent questions and assess comprehension before evaluating retention.

### 4.4. Dental Trauma Knowledge in the Preschool Setting

The distinction between primary and permanent teeth is particularly relevant to preschool teachers. In this cohort, recognition that avulsed primary teeth should not be replanted declined, while endorsement of immediate replantation of an avulsed permanent tooth remained high. International Association of Dental Traumatology guidance treats these situations differently [18,19]. A general instruction to save an avulsed tooth is therefore insufficient unless teachers can identify the relevant dentition and select an appropriate response.

Difficulties with dental trauma knowledge have been reported across educational settings. Sae-Lim and Lim found gaps among preschool teachers in Singapore, including uncertainty about suitable storage media for avulsed permanent teeth [22]. Fittler et al. reported improved responses after dissemination of educational materials in Hungary, although the pre- and post-intervention samples could not be individually linked [23]. The latter study illustrates why improvements in a school population and changes within the same individuals are related but methodologically different observations.

More recent evidence also indicates that gains may weaken over time. In a 2026 single-arm study, Aykanat and Tunc found that teachers’ dental trauma knowledge rose immediately after ToothSOS-based education and declined by one month, while remaining above baseline [24]. Our findings extend the follow-up perspective to a longer interval, but use different items and lack a pre-education assessment; effect sizes cannot be compared directly. Together, these observations support examining retention as a separate educational outcome.

Low-burden resources may help maintain access to emergency information. Lieger et al. found better dental-trauma knowledge in an area exposed to an educational poster campaign, although the design does not isolate a causal poster effect [25]. A scoping review identified several school-based educational approaches and supported continued attention to first-aid education [26]. For preschool settings, practical scenarios should separate primary-tooth avulsion from permanent-tooth avulsion and include recognition, handling and referral decisions. Questionnaire responses alone do not demonstrate the ability to manage an actual injury.

### 4.5. Knowledge and Everyday Practice

Personal preventive practices did not simply mirror endorsement of preventive messages. At 12 months, 61.8% selected regular dental visits as a preventive measure, but only 15.8% reported routine attendance without symptoms. These aggregate proportions cannot identify individual discordance, yet they indicate that approval of a recommendation and reported adherence are different outcomes. The decrease in brushing at least twice daily likewise suggests that educational follow-up should include behaviour as well as knowledge.

This distinction is consistent with Yilmaz et al.’s observation that teachers’ reported oral hygiene behaviours did not closely track their knowledge and attitudes [8]. Qualitative findings from Nigerian teachers also identify time, financial constraints, access and the low priority given to oral health as barriers [27]. The increase in reports of insufficient time in our cohort is compatible with that broader context, but no causal association was tested. Knowledge reinforcement alone may therefore be insufficient to change personal attendance patterns.

The capability, opportunity and motivation framework offers a useful way to formulate future programme components [28]. Education primarily addresses knowledge-related capability, while time, access and institutional support affect opportunities to act. This framework was not used to design or test the original intervention and is offered here as an interpretive model. A revised programme could evaluate these domains separately, rather than treating every unfavourable response as a failure to remember information.

### 4.6. Implications for School and Family Programmes

Maintaining teachers’ knowledge is one component of a wider preventive strategy. School-based programmes can also engage parents, whose daily decisions influence children’s routines. Shan et al. found that a parental workshop improved oral health knowledge initially, but intervention and comparison groups did not differ significantly in knowledge at 12 months or in the change in children’s caries experience [29]. Tahani et al. evaluated an integrated school approach combining educational contacts and home activities [30]. These studies reinforce the need to distinguish the contributions of teachers, families and programme delivery.

The development and validation of the School Oral Health Promotion Program (SOHEPP) video offers a recent example of producing standardised educational material for teachers and adolescents [31]. Such resources could support consistent delivery or later review, but validation of a teaching resource is not proof of sustained learning or clinical effectiveness. For this programme, the next step would be to combine updated content with documented reinforcement and evaluate teacher knowledge, classroom implementation and appropriate child outcomes separately. This would address the gap between favourable questionnaire responses and an oral-health-promoting school environment. Future studies should document the duration and number of educational contacts per teacher and assess whether the planned material was delivered consistently across schools.

### 4.7. Strengths and Limitations

The principal strength is the paired follow-up of 152 teachers after education delivered across 63 schools. Revisiting the same teachers provides a clearer assessment of post-education change than comparisons of independent cross-sectional samples. Presenting stable responses alongside declining ones avoids describing the programme as uniformly successful or unsuccessful and helps identify practical priorities for revision.

Several limitations affect interpretation. The absence of matched pre-education measurements and a concurrent comparison group prevents estimation of initial educational effectiveness and causal attribution of subsequent changes. Only 63.3% of trained teachers contributed paired observations. Teachers who completed both assessments may have differed systematically in knowledge, motivation, or practices from those who were lost to follow-up. Comparable individual-level information for the 70 teachers assessed at 3 months only was unavailable, so the direction and size of this potential selection bias cannot be evaluated; the findings may not represent all teachers who received education. Self-reported practices and repeated questionnaire completion may also be affected by recall, social desirability, and testing effects.

The questionnaire was not validated as a contemporary knowledge scale, and some historical wording requires revision. The analyses involved multiple unadjusted tests and did not account for school-level clustering; some nominally statistically significant findings may therefore be chance observations. Inferential findings should be regarded as exploratory, especially those close to the nominal significance threshold. Individual transition data were unavailable for independent verification of paired tests or estimation of paired confidence intervals. Precise assessment dates, educational dose, delivery fidelity, and other educational exposures during follow-up were insufficiently documented. Future studies should record these features prospectively. Finally, no child-level clinical outcomes or observed teaching and emergency-management behaviours were assessed. These limitations favour an interpretation focused on message-specific follow-up and programme development, rather than proof of clinical benefit.

## 5. Conclusions

Paired follow-up identified changes in teachers’ knowledge, beliefs, and self-reported practices between 3 and 12 months after education. Less frequent endorsement of fluoride-containing products and enamel strengthening, first-tooth cleaning, regular preventive dental visits, and the recommendation not to replant avulsed primary teeth identifies specific topics for updated content and structured reinforcement. Some responses remained relatively stable. Controlled evaluation with matched pre-education measurements is needed to determine whether these refinements improve sustained teacher knowledge, practical implementation, and children’s oral health.

## Figures and Tables

**Table 1:** Characteristics of teachers included in the paired follow-up analysis.

Characteristic	*n*	%
Women	133	87.5
Men	19	12.5
Age 25 years or younger	11	7.2
Age 26–30 years	56	36.8
Age 31–36 years	46	30.3
Age 37 years or older	39	25.7
Has children	112	73.7
Does not have children	40	26.3
Professional experience 0–5 years	23	15.1
Professional experience 6–10 years	52	34.2
Professional experience 11–15 years	50	32.9
Professional experience 16 years or more	27	17.8

*n* = 152. Percentages were calculated from counts and rounded to one decimal place.

**Table 2:** Responses concerning caries and prevention at 3 and 12 months.

Response Endorsed	3 Months *n* (%)	12 Months *n* (%)	Change pp	*p* Value
Inadequate brushing as a caries contributor	150 (98.7)	143 (94.1)	−4.6	0.039
Sweet or acidic foods as a caries contributor	150 (98.7)	147 (96.7)	−2.0	0.375
Absence of regular check-ups as a caries contributor	129 (84.9)	96 (63.2)	−21.7	<0.001
Dental plaque as a caries contributor	128 (84.2)	108 (71.1)	−13.2	<0.001
Oral health affects general health	149 (98.0)	136 (89.5)	−8.6	0.001
Caries-free teeth are possible	143 (94.1)	121 (79.6)	−14.5	<0.001
Problems in primary teeth affect permanent teeth	138 (90.8)	116 (76.3)	−14.5	<0.001
Caries in primary teeth can be treated	149 (98.0)	147 (96.7)	−1.3	0.625
Limiting sugary snacks for caries prevention	145 (95.4)	141 (92.8)	−2.6	0.388
Fluoride-containing dental products for caries prevention	97 (63.8)	61 (40.1)	−23.7	<0.001
Sugar-free chewing gum for caries prevention	113 (74.3)	77 (50.7)	−23.7	<0.001
Toothbrushing for caries prevention	151 (99.3)	144 (94.7)	−4.6	0.039
Dental floss for caries prevention	141 (92.8)	119 (78.3)	−14.5	<0.001
Regular dental visits for caries prevention	128 (84.2)	94 (61.8)	−22.4	<0.001
Encouraging cheese, milk, or yogurt as snacks	128 (84.2)	110 (72.4)	−11.8	0.004

*n* = 152 at each assessment. pp, percentage points; change = 12 months minus 3 months. *p* values are the original unadjusted paired McNemar results. Multiple responses were permitted. Items describe questionnaire responses rather than a validated correctness scale. “Sweet or acidic foods” combines distinct concepts from the original wording. “Fluoride-containing products” refers to dental-product use, not ingestion. Percentages and changes were calculated from counts before rounding.

**Table 3:** Early oral care, fluoride beliefs and dental injury responses.

Response Endorsed	3 Months *n* (%)	12 Months *n* (%)	Change pp	*p* Value
Begin cleaning when the first tooth erupts	141 (92.8)	115 (75.7)	−17.1	<0.001
First dental examination no later than age 1 year	130 (85.5)	116 (76.3)	−9.2	0.013
Fluoride strengthens enamel	126 (82.9)	101 (66.4)	−16.4	<0.001
Fluoride is nutritive for teeth and bones [a]	118 (77.6)	84 (55.3)	−22.4	<0.001
Fluoride acts against oral bacteria	123 (80.9)	92 (60.5)	−20.4	<0.001
Fluoride can improve initial carious lesions	117 (77.0)	86 (56.6)	−20.4	<0.001
Start fluoride toothpaste at approximately age 3 years [a]	98 (64.5)	83 (54.6)	−9.9	0.028
Children should not use fluoride toothpaste [a]	31 (20.4)	45 (29.6)	+9.2	NR
Oral injuries should be assessed promptly	141 (92.8)	133 (87.5)	−5.3	0.021
An avulsed primary tooth should not be replanted	127 (83.6)	107 (70.4)	−13.2	<0.001
An avulsed permanent tooth should be replanted immediately	139 (91.4)	136 (89.5)	−2.0	0.508
Non-nutritive sucking habits affect the teeth and jaws	147 (96.7)	147 (96.7)	0.0	1.000

*n* = 152. pp, percentage points; NR, paired test not reported. *p* values are the original unadjusted McNemar results. [a] Historical questionnaire beliefs are not classified as correct contemporary preventive advice. Reduced agreement with an incorrect or ambiguous statement is not evidence of loss of correct knowledge. No practical trauma-management assessment was performed.

**Table 4:** Self-reported personal oral health practices and attitudes.

Practice or Belief	3 Months *n* (%)	12 Months *n* (%)	Change pp	*p* Value
Brushing at least twice daily	126 (82.9)	111 (73.0)	−9.9	0.001
Brushing for 2–3 min	109 (71.7)	97 (63.8)	−7.9	0.081
Replacing the toothbrush at fixed intervals [a]	138 (90.8)	122 (80.3)	−10.5	0.004
Performing interdental cleaning	120 (78.9)	108 (71.1)	−7.9	0.088
Routine dental visits without pain or other problems	25 (16.4)	24 (15.8)	−0.7	1.000
Belief that dentists sufficiently prioritize prevention	92 (60.5)	115 (75.7)	+15.1	0.001

*n* = 152. pp, percentage points. *p* values are the original unadjusted paired McNemar results. [a] The category combines replacement every 3 months, every 6 months and annually; it does not denote adherence to one recommended interval. All practices were self-reported.

**Table 5:** Reported reasons for avoiding routine dental attendance or disliking dental visits.

Reason	3 Months *n* (%)	12 Months *n* (%)
Fear	23 (15.1)	14 (9.2)
Cost	25 (16.4)	16 (10.5)
No nearby clinic	3 (2.0)	12 (7.9)
Lack of time	30 (19.7)	54 (35.5)
No particular reason	71 (46.7)	55 (36.2)
Missing response	0 (0.0)	1 (0.7)

The denominator is all 152 paired teachers. One response was missing at 12 months. No paired significance tests were reported; differences are descriptive.

## Data Availability

De-identified data supporting the findings of this study are available from the corresponding author upon reasonable request. The data are not publicly available to protect participant confidentiality.

## Abbreviations

SOHEPP, School Oral Health Promotion Program; SPSS, Statistical Package for the Social Sciences.
